# The Effect of Spot Size Combination Mode on Ablation Morphology of Aluminum Alloy by Millisecond-Nanosecond Combined-Pulse Laser

**DOI:** 10.3390/ma11081419

**Published:** 2018-08-13

**Authors:** Bo-Shi Yuan, Ye Zhang, Wei Zhang, Yuan Dong, Guang-Yong Jin

**Affiliations:** The Key Laboratory of Jilin Province Solid-State Laser Technology and Application, Chang Chun University of Science and Technology, Changchun 130022, China; yuanboshi1986@163.com (B.-S.Y.); zhangye84829@163.com (Y.Z.); a5371863@163.com (W.Z.); laser_dongyuan@163.com (Y.D.)

**Keywords:** spot size combination mode, ablation morphology, combined pulse laser, laser processing

## Abstract

Ablation morphology affects the quality of laser processing. Therefore, the control of ablation morphology is very important. The influence of spot size combination mode on the ablation morphology of aluminum alloy is studied for the first time. Experimental results show that when the nanosecond laser spot is larger, the ablation morphology looks like a bowl-shape, and there is little solidification near the edge. When the nanosecond laser spot is smaller, the shape of the ablation morphology is similar to a hole, and the protuberance is formed near the edge of the cavity. Through the analysis and simulation of the physical model, the physical mechanism, which describes the influence of the spot size combination mode on the molten pool, is discussed. The research results of this paper have important guiding significance for the control of laser processing effect.

## 1. Introduction

The Al alloy is one of the most important metal materials in industry. It has excellent electrical conductivity and thermal conductivity, normal temperature and the low temperature plasticity, corrosion resistance, and is the metal material that is favored by of lots of industries, such as electronics, automobile, and aircraft, etc. The ms laser belongs to the hot processing sources in laser processing. The processing efficiency is higher than those of a short-pulse laser and ultrashort-pulse laser; however, the processing effect is not satisfactory. There are problems of the heat-affected zone, surface splashing, etc. In recent decades, the method of laser combination has attracted considerable attentions from not only the Laser-Induced Breakdown Spectroscopy (LIBS) [1,2], but also the practical laser processing industrial applications [3,4,5,6,7,8,9,10]. In the field of laser drilling, cutting and welding, the laser processing technology has become an indispensable choice due to its advantages of noncontact, precise, no consumption, high flexibility, easy integration, automation and reproducible technique [11].

In the research of the combined-laser in laser processing application, the primary concern of the research is faster efficiency and better quality. J. A. Fox et al. [3] firstly combined a 25 ns pulse laser with a CO_2_ continuous wave laser, the irradiation time that is necessary for the penetration of 20-mil steel targets decreased by more than a factor of two. The time that is required for the beam to penetrate the target is shorter, and the hole of the resolidified materials is significantly clearer. C. Lehane et al. [4] investigated the laser drilling of stainless steel by combining a 3.5-ms laser with a 0.15-ms laser. The influence of the interval time between the two pulses on the drilling efficiency was studied and an optimal delay time was revealed. A. C. Forsman [5] and X. D. Wang et al. [6] also verified that the material removal rates and drilling quality could be improved by applying the double-pulse technique with two nanosecond-pulse laser separated by several tens of nanoseconds. Based on an experiment, the interaction of the combined laser with the material was explained. C. Hartmann et al. [7] investigated on laser micro ablation of metals using ns-multi-pulses, and the process was characterized in terms of ablation rate, ablation geometry, the dynamic of plasma expansion, and plasma parameters. M. Brajdic and K. Walther et al. [8,9] analyzed the laser drilling of deep holes in stainless steel by combining a 0.5 ms pulse laser and a 17-ns pulse laser. The drilling efficiency was improved by the spatially and temporally superposed radiation of the two lasers. Z. Wang et al. [10] drilled holes with double-pulsed (ms and ns) laser and analyzed the influence of the time interval, the energy and the pulse width of ms laser energy on the hole geometry, and the material removal.

The previous has done much relevant research and used the delay time effect to improve laser processing efficiency and quality. However, there is another special laser parameter in the combined laser, that is, the spot size combination mode of the two laser beams. The spot size could affect the physical model and the mechanism on the irradiated area of the target. The influence of spot size on the processing of laser interaction with matter has also been studied extensively. H. C. Pant et al. [12] studied the effect of the laser spot size on laser light absorption in laser induced plasmas from solid targets. It was found that laser focal spot size on the target has an effect on the optical energy balance in laser produced plasmas. M. Eyett et al. [13] observed the influence of the beam spot size on ablation rates in pulsed-laser processing, and considered that the effect is due to changes in the material transport, which depend on the size of the region being processed. Diaspro, A., et al. [14] found the spot size strongly influence the ablation efficiency in silicon when drilling holes with a diameter that is smaller than 200 μm while using ns laser pulses. These findings are explained in terms of plume shielding effect. Li, Xingwen, et al. [15] used fast photography and optical emission spectroscopy to investigate the laser produced copper plasmas of different spot sizes in the air. The results showed the spot size strongly influenced the laser produced plasma dynamics, a large spot size resulting in hemispherical structure and a small spot size in a stream-like shape of the plasma plume. F.Z. Dai et al. [16] used different peak pressure with a different laser spot diameter to the surface of pure Al, and researched the effect of laser spot size on the residual stress field of pure Al that is treated by laser shock processing. S. S. Harilal et al. [17] investigated the role of spot size on plume morphology during ultrafast laser ablation of metal targets. Their results show that the spatial features of plumes are strongly dependent on the focal spot size, and the morphological changes in the plume with spot size are independent of laser pulse width. As mentioned above, the spot size has an important effect on the laser action; however, there is no relevant report about the influence of spot size on ablation morphology in the combined laser.

In the present study, the influence of spot size combination mode on the ablation morphology of aluminum alloy is studied for the first time. The results show significant differences in the ablation morphology under different spot size combination modes. It is considered that the change of spot size combination mode not only affects the expansion of the plasma plume, but also changes the flow form of the melt in the molten pool. The influence of spot size combination mode on the ablation morphology was analyzed to reveal the underlying physical mechanism.

## 2. Experimental Method and Materials

### 2.1. Experimental Method

**Figure 1:**
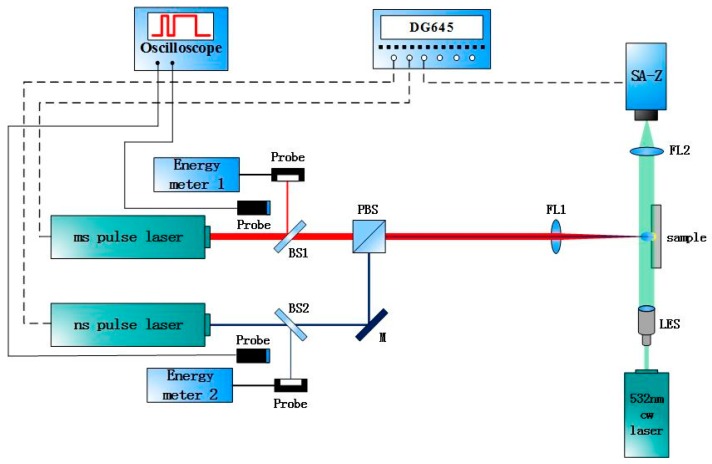
Experimental setup for ms-ns pulse combination laser processing of aluminum alloy.

The experimental setup is shown in Figure 1. The experimental system consists of three parts, including laser emission and monitor, plasma expansion measurement, and surface morphology measurement.

Laser emission and monitor includes millisecond pulse laser, Melar-100 (Beamtech, Beijing, China), nanosecond pulse laser, MQU-2000 (Beijing ZK Laser Co., Ltd, Beijing, China) energy detector, oscilloscope, spectroscope (BS1, BS2), mirror(M), focusing lens(FL1), etc. The wavelengths of ms laser and ns laser are both 1064 nm, and the pulse widths are 10 ns and 1 ms, respectively. Both lasers pass through the focus lens (f = 500 mm) and locate on the same point of the sample surface. The high-speed shadowgraphy technique is used to measure the plasma plume. It contains 532 nm continuous laser, MSL-III-532 (Changchun, Jilin, China), extender lens, focus lens (f = 50 mm), and high-speed camera, Fastcam SA-Z (Photron. Ltd, Shanghai, China). In the experimental setup, the exposure time is 1/6,300,000 s and the frames per second is 200,000 fps. The expansion characteristics of the plasma plume are observed by the shadow on the surface. Surface morphology is measured by Infinite Focus System G4 (Alicona, Graz, Austria). The pulse delay is controlled by the Digital delay/ pulse generator, DG645 (Stanford Research Systems, Stanford, USA).

### 2.2. Materials

In the experiment, the sample is fixed on a three-dimensional displacement stage, and the experiment environment is at one atmosphere. The material of samples that is used in the experiment is 7A04 aluminum alloy with a size of 25 × 25 × 3 mm. It belongs to Al-Zn-Mg-Cu series ultrahigh strength aluminum alloy.

## 3. Experimental Results

In the experiment, the pulse delay is an important factor. According to the rule of millisecond single pulse laser irradiating on the aluminum alloy, the thermal effect enhances with the increase of the duration of the millisecond laser. In order to obtain the larger molten pool and to obtain the ideal ablation morphology, the pulse delay is selected as 800 μs, according to the results of the repeated experiment. In the experimental setup, the pulse delay is the time delay when the ns laser starts to work after the ms laser starts. The ms laser pulse width is 1 ms, so the interaction between the ns laser and the target occurred during the duration of the ms laser pulse width.

In this research, the millisecond pulsed laser spot radius is marked as $r_{ms}$, and the nanosecond pulsed laser spot radius is marked as $r_{ns}$. The different ablation morphology and plasma plume expansion were researched under the conditions of between $r_{\mathrm{ms}}<r_{\mathrm{ns}}$ and $r_{\mathrm{ms}}>r_{\mathrm{ns}}$ The main laser parameters are shown in Table 1.

### 3.1. Analysis of Ablation Morphology

**Figure 2:**
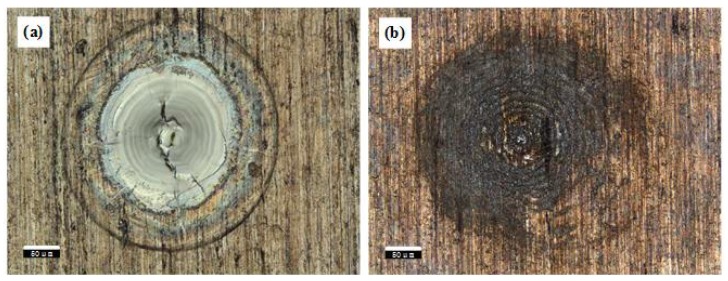
The ablation morphology under different pulse width laser (**a**) ms pulse laser (**b**) ns pulse laser.

In Figure 2, it showed photos of sample surface ablated by only the ms-pulsed laser and by only the ns-pulsed laser. It can be observed there is a molten pool formed by the ms laser. The colour of the surface that is irradiated by ns laser is different, but there is no molten pool.

**Figure 3:**
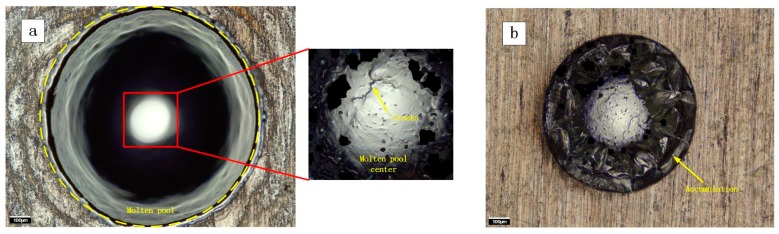
The ablation morphology under different spot size combination modes. (**a**) the ns spot is larger (**b**) the ms spot is larger.

From the ablation morphology of the aluminum alloy, the influence of spot size combination mode on the ablation morphology can be observed intuitively. Figure 3 is the aluminum alloy morphology that is measured in two different spot size combination modes. Figure 3a is the result of the ns laser spot larger. The center of the ablation morphology is a melting area, and the color is silver gray because of the oxidation of the molten substance. There are many cracks in the center of the melting area. The reason for these cracks is that the molten pool surface is rapidly cooled and solidified from high temperature, and shrinkage stress is greater than the plastic deformation stress during solidification [18]. There is an outer edge that distinguishes the melting area from the other area. The surface modification is happened in the other area by ns laser. Figure 3b is the result of the ms laser spot larger. In contrast to the morphology of Figure 3a, the melting area presents silver grey in the center of the oxidation reaction. On the periphery of the oxidized area, the melt forms accumulation. There is a wrinkled appearance on the surface of the accumulation. The accumulation of these melts may be due to the extrusion under mechanical action. In addition, as shown in the ablation morphologies, there is no slagging phenomenon. So, combined laser has certain advantages in laser processing.

**Figure 4:**
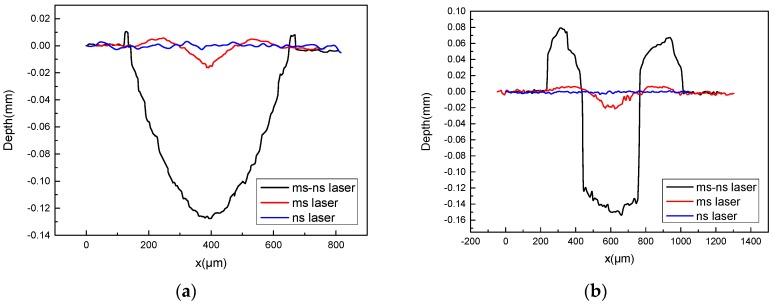
The profile of ablation morphologies under different spot size combination modes (**a**) the ns spot is larger (**b**) the ms spot is larger.

Figure 4 shows a more intuitive comparison of the profile of ablation morphologies in both conditions. In case 1, the millisecond laser spot can be covered by the nanosecond laser. A cavity looks like a bowl on the surface, which has been caused by ablation. There is little molten material accumulated on the edge. It is caused by the solidification of the molten material at the edge during the spray. But, in case 2, when the nanosecond pulse laser spot is smaller, the shape of the ablation morphology has obviously changed. The internal side of the cavity is steep, and the cavity forms a hole. The accumulation is more on the edge of the hole. The formation of protuberances may be caused by the flow of the melt.

The experimental data was further measured on the ablation profile of aluminum alloys, and the quantitative analysis was performed through the data to determine the main factors affecting the morphology change.

Analyzed from the data in Table 2, there is no obvious change in depth under different laser parameters. Because the depth of ablation is mainly influenced by laser energy density and pulse delay. In the experiment, the pulse delay is constant, and the energy density determines the size of the molten pool. So, when the energy density is approximate, the depth is more or less. The spot size has little effect on the depth of the ablation.

In case 2, the radius of the hole is smaller than the ms laser radius, but it is approximate to the ns laser radius. The removal volume from the hole is less than the volume of accumulation around the hole. It may be due to the phase transition of the aluminum alloy. The structure becomes loose when the molten material resolidified around the hole.

For the difference of the ablation morphologies in different spot size combination mode, the main reason is that the migration form of the melt has changed. Before the ns laser beginning, the aluminum alloy has formed a molten pool under the irradiation of ms laser. At the moment, the physical models of the aluminum alloy under ms laser action are the same. However, for the ns laser, the physical models under the two spot combination modes are quite different. Because of the difference in spot size, the boundary conditions of ns laser irradiation area have changed. When the ns laser spot is the larger, the ns laser spot can cover both the entire molten pool and the solid aluminum alloy outside the molten pool. The boundary of the ns laser irradiated area is solid. When the ns laser spot is the smaller, the ns laser only irradiates on the molten pool. The boundary of ns laser irradiated area is liquid. As the boundary conditions change, the movement form of the melt in the pool also changes. The melt flow in the molten pool is determined by the surface tension and recoil pressure. The recoil pressure is the mechanical effect that is caused by the plasma plume and shock wave produced by the ns laser on the target surface. Therefore, the study of the characteristics of plasma plume and shockwave on the target surface under different laser spot size combination modes is helpful to analyze the physical process and mass migration form.

### 3.2. The Evolution Characteristics of The Plasma Plume

To further investigate the effect of the spot size combination mode on the ablation process, the method of high-speed shadowgraph was used to observe the evolution characteristics of the plasma plume on the target surface.

Analyzing from shadowgraphs, the plasma plume and shockwave were generated on the surface of the target under both spot size combination modes. A small amount of plasma is generated during the millisecond laser irradiation. These plasmas may be due to impurities in the alloy. Under the action of ns laser, the plasma plume on the target surface rapidly expands and shock waves are generated [19].

**Figure 5:**
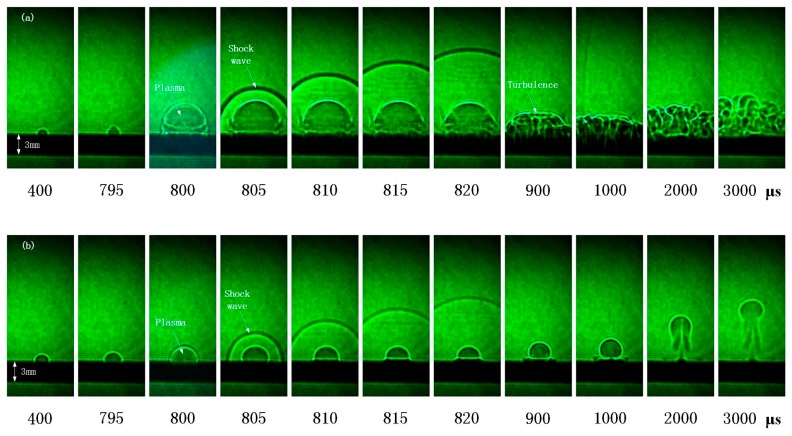
The evolution characteristics of the plasma plume under different spot size combination modes (**a**) the ns spot is larger (**b**) the ms spot is larger.

When the nanosecond laser spot is larger, as shown in Figure 5a, the expansion volume of plasma plume becomes larger and splattering occurs on the surface of the target under the action of the nanosecond laser. When the nanosecond laser stops, the plasma plume decays. The strong turbulence occurs on the surface of the target [20,21,22]. In Figure 5b, when the nanosecond laser spot is smaller, the volume of the plasma plume is also relatively smaller, and no splattering occurs on the surface of the target. When the nanosecond laser stops, the plasma plume changes slowly. It can be observed that the plasma plume expands against the laser incident direction without radial expansion. It indicates that the plasma plume continues to expand in the laser direction due to the absorption of the millisecond laser energy. Another important reason is that the plasma is not too much, and the subsequent millisecond laser energy is enough to maintain expanding. In addition, by associating with the ablation morphology, the ablation morphology is similar to the shape of the molten pool when the molten pool splashed violently. The radius of the ablation morphology is also related to the plasma plume radius. The larger the plasma plume radius, the larger the ablation radius.

## 4. Analysis and Discussion

### 4.1. Mechanism Analysis

**Figure 6:**
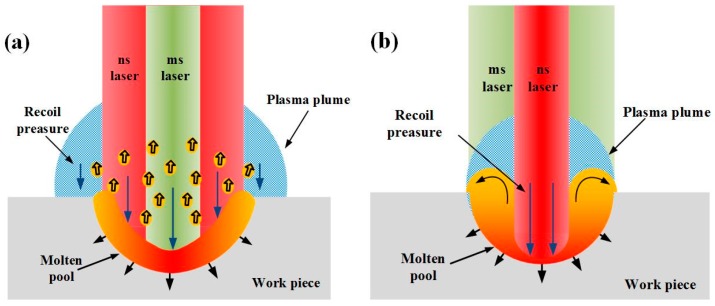
Schematic illustration of the laser ablation (**a**) the ns spot is larger (**b**) the ms spot is larger.

In Figure 6a, when the nanosecond laser spot is larger, the entire surface of the molten pool is under the action of the nanosecond laser. With the temperature increasing, the surface tension becomes smaller, and the recoil pressure becomes larger due to the expansion of the plasma plume and shock wave. At the moment, since the nanosecond laser covers the entire molten pool, the boundary condition of the molten pool changed. The solid-phase aluminum alloy confines the melt flowing. In the condition that the surface tension is less than the recoil pressure, the melt splashes.

In Figure 6b, when the nanosecond laser spot is smaller, the nanosecond laser only irradiates on the central area of the molten pool rather than the whole molten pool. The effect of the nanosecond laser makes the surface tension of the melt in the central area reduces and the recoil pressure increase. The fluid in the molten pool flows from the low surface tension to the high surface tension. Therefore, the melt flows from the center to the outside, and the accumulation is formed [23,24].

### 4.2. Physical Model and Analysis

**Figure 7:**
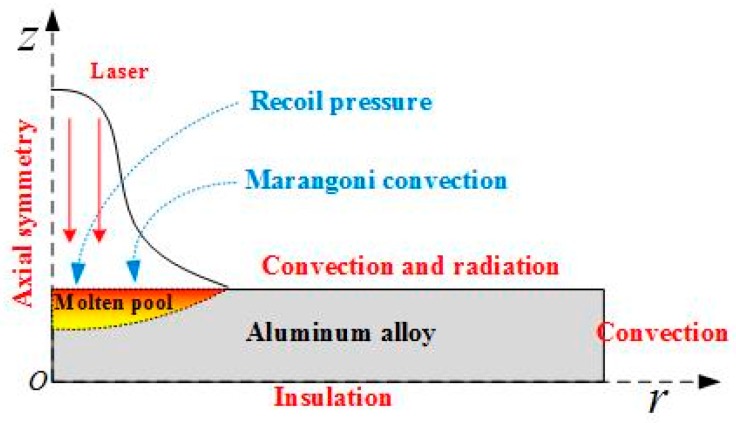
Schematic illustration of the physical model.

To describe the influence of the spot size combination mode on the molten pool, a two-dimensional (2D) axial symmetry physical model (Figure 7) has been developed based on the following assumptions [25,26]:

- The material is considered to be isotropic and homogenous.
- The flow of liquid metal in the molten pool is treated as incompressible Newtonian laminar flow.
- The thermo-physical properties related to aluminum alloy depend on the temperature in solid and liquid phase.

In order to evaluate the levels of temperature that are reached in the process and to obtain characteristics of molten pool, conservation of energy (Fourier’s Law), mass (continuity), and momentum (Navier-Stokes) equations are solved. The governing equations are shown below,

(1) $$\rho C_{p}\left[\frac{\partial T}{\partial t}+\vec{u}\cdot \nabla T\right]-\nabla \cdot \left(k\nabla T\right)=Q$$

(2) $$\rho \frac{d\vec{u}}{dt}=-\nabla p+\mu \nabla^{2}\vec{u}+F_{v}$$

(3) $$\nabla \cdot \vec{u}=0$$

where, ρ is the density of the material $\left(kg/m^{3}\right)$, p is the pressure $\left(N/m^{2}\right)$, $\vec{u}$ is the molten metal velocity (m/s), $C_{p}$ is the specific heat of the material (J/kgK), $F_{v}$ is the body force (N), μ is the dynamic viscosity (Pa⋅s), k is the thermal conductivity (W/mK), and T is the absolute temperature (K).

(4) $$-k\nabla T=I_{0}\alpha e^{-\frac{2r^{2}}{r_{0}^{2}}}$$

In Equation (1), because the laser absorption depth of metal is small, the laser intensity can be defined as a separate boundary heat flux Equation (4) to instead of using a source term. Where $I_{0}$ is the laser energy density at the center $\left(J/cm^{2}\right)$, α is the absorption coefficient of aluminum alloy for 1064 nm wavelength, and $r_{0}$ is the laser spot radius. The laser distribution is Gaussian.

At the top and side boundary, convection boundary condition Equation (5) is used, further at top boundary radiation Equation (6) due to high intensity laser spot is also considered, the bottom boundary Equation (7) is assumed to be insulated.

(5) $$-k\nabla T=h\left(T-T_{0}\right)$$

(6) $$-k\nabla T=\varepsilon \sigma \left(T^{4}-T_{0}^{4}\right)$$

(7) ∇T=0

where h is convective heat transfer coefficient, ε is surface radiation coefficient, and σ is Stefan-Boltzmann constant.

Moreover, the molten metal is considered to be laminar and the dynamic viscosity of solid is assumed to be very high. Before vaporization, only surface tension and gravity forces act on the molten metal, the effects of surface tension is implemented by using a weak constraint term on the top boundary, which is dependent on the tangential velocity of the melt.

(8) $$\sigma_{n}=\kappa \gamma \vec{n}$$

Further, as vaporization temperature is reached, a recoil pressure acts on the vapor liquid interface, whose expression is given by

(9) $$R_{p}=0.54\times P_{a}\times \exp\left(\frac{L_{v}\left(T-T_{v}\right)}{RTT_{v}}\right)\times \exp\left(-\frac{2r^{2}}{r_{0}^{2}}\right)$$

where κ is curvature, γ is surface tension coefficient, $P_{a}$ atmospheric pressure, $L_{v}$ is latent heat of vaporization, $T_{v}$ is vaporization temperature, and R is universal gas constant. The spatial distribution of laser intensity that makes sure that the recoil pressure acts inside the irradiated area instead of whole boundary. The recoil pressure is exerted using an open boundary condition also the recoil pressure is effective only on those regions where the temperature has reached its vaporization point. In addition, the surface is also under the recoil pressure that is caused by the plasma plume and the laser support absorption wave (LSAW), $P_{plasma}$ and $P_{LSAW}$.

In the molten pool, the temperature induced thermos-capillary convection (Marangoni effect). The Marangoni effect stresses in the melt redistribution process. Especially, if there is no spray in the molten pool, the Marangoni effect plays a very important role. In the calculation model, the Marangoni effect is applied at the top surface while using a weak constraint.

(10) $$\sigma_{t}=\frac{\partial \gamma }{\partial T}\vec{\nabla }T\cdot \vec{t}$$

where $\vec{\nabla }T$ is the temperature gradient along the surface.

**Figure 8:**
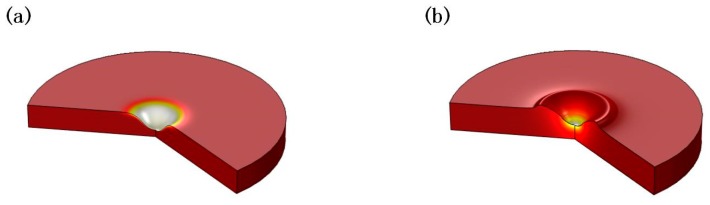
The simulation results of ablation morphology (**a**) the ns spot is larger (**b**) the ms spot is larger.

Based on the physical model, the ablation morphology due to different laser spot size combination modes was simulated (Figure 8). The nature of the cavity is obviated. When the nanosecond laser spot is larger, the recoil pressure covers the entire molten pool. The surface tension of the material becomes smaller as the temperature increases. The surface of the molten pool is subjected to high recoil pressure. In the molten pool, the Marangoni effect is very weak, but the ablation and the splashing have become dominant. A large amount of material removal makes the ablation morphology appear, as shown in Figure 8a. When compared with the experiment, the laser ablation morphology is reasonable under this spot size combination mode.

When the nanosecond laser spot is smaller, temperature and recoil pressure change greatly in the central region of the nanosecond spot. There is a greater temperature gradient in the molten pool. This makes the Marangoni effect even more important [27]. With the help of the Marangoni effect, the melt flows from the center to the edge and it formats protuberance near the edge of the cavity. The simulation result also shows a significant protuberance. It is in agreement with the experimental results. When compared with the experiment, the cavity is not as steep, as shown in Figure 4b. But, the simulation results verify theoretical analysis of the formation of protruding.

It can be concluded from above qualitative analysis that the ablation morphology can be influenced by the laser spot size combination mode. The laser spot size changes the boundary conditions of the physical model, thus affecting the process of multiphysics, which changes the ablation morphology. The recoil pressure and the Marangoni effect have an important influence on the distribution of the melt motion.

## 5. Conclusions

In summary, through the analysis of the ablation morphology of the aluminum alloy irradiated with the millisecond-nanosecond combined-pulse laser, the spot size combination mode plays a key role in the formation of the ablation morphology. The laser spot size combination mode affects the thermal and flow process on the distribution of the melt. When the nanosecond laser spot radius is larger, ablation and splashing of melt are more likely to occur. The morphology is similar to the shape of a bowl-shaped cavity, and the re-solidification on the cavity edge is little. When the nanosecond laser spot radius is smaller, the cavity is similar to a hole, and the Marangoni effect plays an important role on the formation of protuberance near the edge of the cavity. The research results of this paper have important guiding significance for the control of the laser processing effect.

## Figures and Tables

**Table 1:** Millisecond-nanosecond combined-pulse laser parameters variable control.

Parameter Name (Unit)	Case 1	Case 2
Spot size combination mode	$r_{\mathrm{ms}}<r_{\mathrm{ns}}$	$r_{\mathrm{ms}}>r_{\mathrm{ns}}$
Millisecond laser spot radius (mm)	0.24	0.38
Millisecond laser energy density (J/cm^2^)	4382.76	4484.86
Nanosecond laser spot radius (mm)	0.65	0.22
Nanosecond laser energy density (J/cm^2^)	112.36	116.32

**Table 2:** Comparison of ablation morphology data of aluminum alloy.

Spot Size Combination Mode	Depth (μm)	Radius (μm)	Volume (μm^3^)
$r_{\mathrm{ms}}<r_{\mathrm{ns}}$	125.37	275.65	9.6 × 10^6^
$r_{\mathrm{ms}}>r_{\mathrm{ns}}$	149.24	175.33 (hole) 386.45 (accumulation)	1.22 × 10^7^ (hole) 2.21 × 10^7^ (accumulation)
